# Gastric Hamartomatous Inverted Polyp: A Rare Subepithelial Lesion With Unusual Endoscopic Ultrasonographic Features

**DOI:** 10.7759/cureus.119499

**Published:** 2026-11-11

**Authors:** Chorouq Mehdaoui, Claire Billioud, Tanguy Fenouil

**Affiliations:** 1 Hepatogastroenterology, University Hospital Mohammed VI of Oujda Morocco, Oujda, MAR; 2 Gastroenterology, Hôpital de la Croix-Rousse, Lyon, FRA; 3 East Multisite Institute of Pathology, Hospices Civils de Lyon, East Hospital Group, Bron, FRA; 4 Lyon East Faculty of Medicine, Claude Bernard Lyon 1 University, Lyon, FRA; 5 Cancer Research Center of Lyon, Claude Bernard Lyon 1 University, Lyon, FRA

**Keywords:** endoscopic ultrasonography, gastric hamartomatous inverted polyp, gastric lipoma, gastric subepithelial lesion, histopathology

## Abstract

Gastric hamartomatous inverted polyp (GHIP) is a rare benign gastric lesion that may mimic other subepithelial tumors, making preoperative diagnosis challenging. We report the case of a 61-year-old man investigated for persistent epigastric pain, unintentional weight loss, and altered bowel habits. Upper gastrointestinal endoscopy revealed a well-circumscribed subepithelial lesion in the gastric fundus, initially suspected to be a gastrointestinal stromal tumor (GIST). Endoscopic ultrasonography (EUS) demonstrated a 25 mm, predominantly hyperechoic submucosal lesion with mild internal heterogeneity and small anechoic areas, favoring a gastric lipoma. Endoscopic ultrasound-guided sampling and deep bite-on-bite biopsies revealed dilated and architecturally distorted fundic-type glands within the submucosa, without cytological atypia. Immunohistochemical staining was positive for pepsinogen and MUC6, supporting the diagnosis of GHIP. This case highlights the importance of considering GHIP in the differential diagnosis of atypical hyperechoic gastric subepithelial lesions and emphasizes the complementary roles of EUS and histopathological examination.

## Introduction

Gastric hamartomatous inverted polyps (GHIPs) are rare benign gastric lesions characterized by the proliferation of gastric glands into the submucosa, often associated with cystic dilatation and intervening smooth muscle bundles [1,2]. GHIPs have been reported as solitary lesions in patients without an underlying hereditary polyposis syndrome [3]. Their pathogenesis remains incompletely understood. One proposed mechanism involves repeated mucosal injury and erosion, leading to disruption of the muscularis mucosae and subsequent displacement of gastric glands into the submucosa [3]. However, the occurrence of these lesions in patients without a history of gastric surgery or other predisposing conditions suggests that additional pathogenic mechanisms may be involved [4]. The clinical presentation of GHIPs is generally nonspecific, and these lesions are discovered incidentally during upper gastrointestinal endoscopy [5]. Their diagnosis remains challenging because of their variable endoscopic and endosonographic appearance, frequently mimicking other gastric subepithelial lesions, including gastrointestinal stromal tumors (GISTs) and lipomas [6]. Endoscopic ultrasonography (EUS) plays an important role in characterizing gastric subepithelial lesions by determining their layer of origin, echogenicity, internal architecture, and relationship to adjacent structures. Furthermore, EUS-guided tissue acquisition may provide histopathological material for establishing a definitive diagnosis [6]. Although GHIPs are generally benign, cases of associated epithelial dysplasia and gastric adenocarcinoma have been reported [3,7]. We report a case of GHIP presenting as a predominantly hyperechoic gastric subepithelial lesion, initially suspected to be a GIST on conventional endoscopy and subsequently interpreted as a possible lipoma (EUS), highlighting the diagnostic value of histopathological examination and immunohistochemical analysis in distinguishing GHIPs from other gastric subepithelial lesions.

## Case presentation

A 61-year-old man with a medical history of chronic obstructive pulmonary disease, dyslipidemia, coronary atherosclerosis, and previous transurethral resection of the prostate had recently been hospitalized for an extraperitoneal bladder perforation associated with urinoma, which was successfully managed conservatively. He was referred for the evaluation of persistent epigastric pain associated with anorexia, deterioration in general condition (Eastern Cooperative Oncology Group performance status of 2), and unintentional weight loss of approximately 5 kg over four months. He also reported altered bowel habits, alternating between diarrhea and constipation, without overt gastrointestinal bleeding. His family history was notable for colorectal cancer in his father.

Laboratory investigations revealed mild normocytic anemia (hemoglobin, 11.7 g/dL; mean corpuscular volume, 93 fL), mild leukocytosis (11.42 x 10^9^/L), and an elevated C-reactive protein level (79.3 mg/L), while renal function and serum electrolytes were largely preserved. The main laboratory findings are summarized in Table 1.

**Table 1:** Laboratory findings at presentation GFR: glomerular filtration rate

Laboratory parameter	Patient value	Reference range
Hematological parameters		
White blood cell count (x10^9^/L)	11.42	4-10.0
Hemoglobin (g/dL)	11.7	13.0-17.0
Mean corpuscular volume (fL)	93	80-100
Platelet count (x10^9^/L)	310	150-400
Inflammatory marker		
C-reactive protein (mg/L)	79.3	<5
Renal function		
Urea (mmol/L)	6.0	2.8-8.1
Creatinine (µmol/L)	56	59-104
Estimated GFR (mL/min/1.73m²)	>90	>90
Sodium (mmol/L)	4.3	3.4-4.5
Potassium (mmol/L)	4.3	3.4-4.5

Contrast-enhanced abdominal computed tomography (CT) demonstrated a well-circumscribed soft-tissue lesion in the gastric fundic region, measuring approximately 19 mm in diameter on axial imaging (Figure 1).

**Figure 1:**
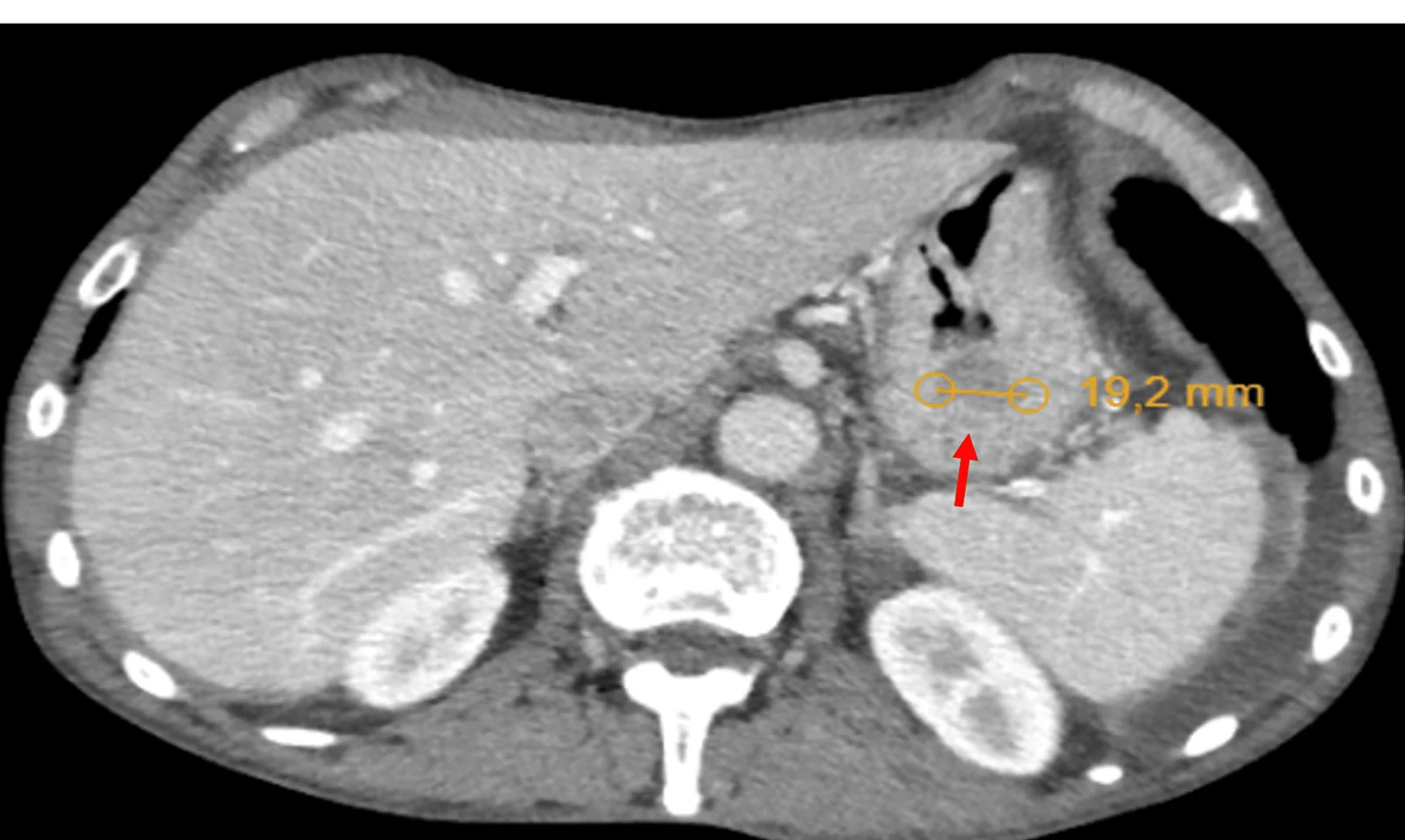
Abdominal CT showing a gastric fundic lesion CT: computed tomography Axial abdominal CT image demonstrating  a well-defined soft tissue lesion measuring  approximately 19.2 mm (orange arrow), located in the gastric fundus (red arrow)

Given the persistence of epigastric symptoms, unintentional weight loss, altered bowel habits, and a family history of colorectal cancer, bidirectional gastrointestinal endoscopy was performed to investigate a possible underlying gastrointestinal malignancy. Colonoscopy was unremarkable, whereas upper gastrointestinal endoscopy revealed a well-circumscribed, broad-based subepithelial protrusion arising from the anterior wall of the gastric fundus, approximately 10 cm distal to the gastroesophageal junction. The lesion was covered by smooth, normal-appearing mucosa without ulceration. Narrow-band imaging (NBI) showed no obvious mucosal or vascular irregularities (Figure 2). Despite its soft, cushion-like appearance, the lesion initially raised suspicion for a GIST.

**Figure 2:**
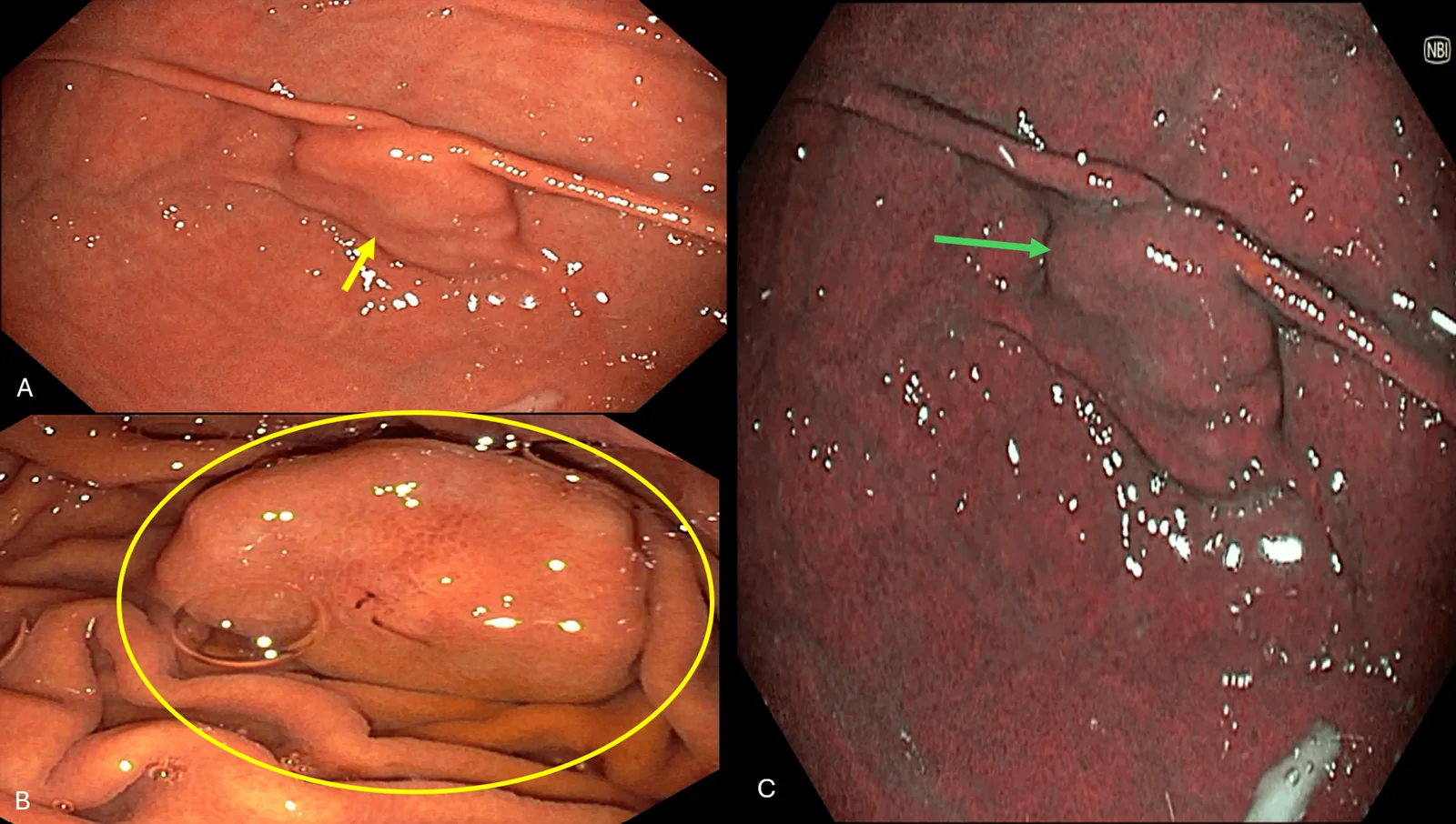
Endoscopic appearance of the gastric subepithelial lesion (A+B) Conventional white-light endoscopy showing a well-circumscribed, broad-based subepithelial protrusion in the gastric fundus, covered by smooth, normal-appearing mucosa without ulceration (yellow arrow in A and yellow circle in B). (C) Narrow-band imaging (NBI) showing no obvious mucosal surface or vascular irregularities (green arrow)

EUS showed a well-defined, predominantly hyperechoic lesion measuring approximately 25 mm, arising from the third layer of the gastric wall (submucosa), with mildly heterogeneous internal echotexture and small hypoechoic areas (Figures 3A-3B). Color Doppler examination demonstrated no significant internal vascularity (Figure 3C), while the underlying muscularis propria remained preserved. These findings were less suggestive of a GIST and initially favored a gastric lipoma, although the internal heterogeneity and small hypoechoic areas were atypical for a conventional lipoma.

**Figure 3:**
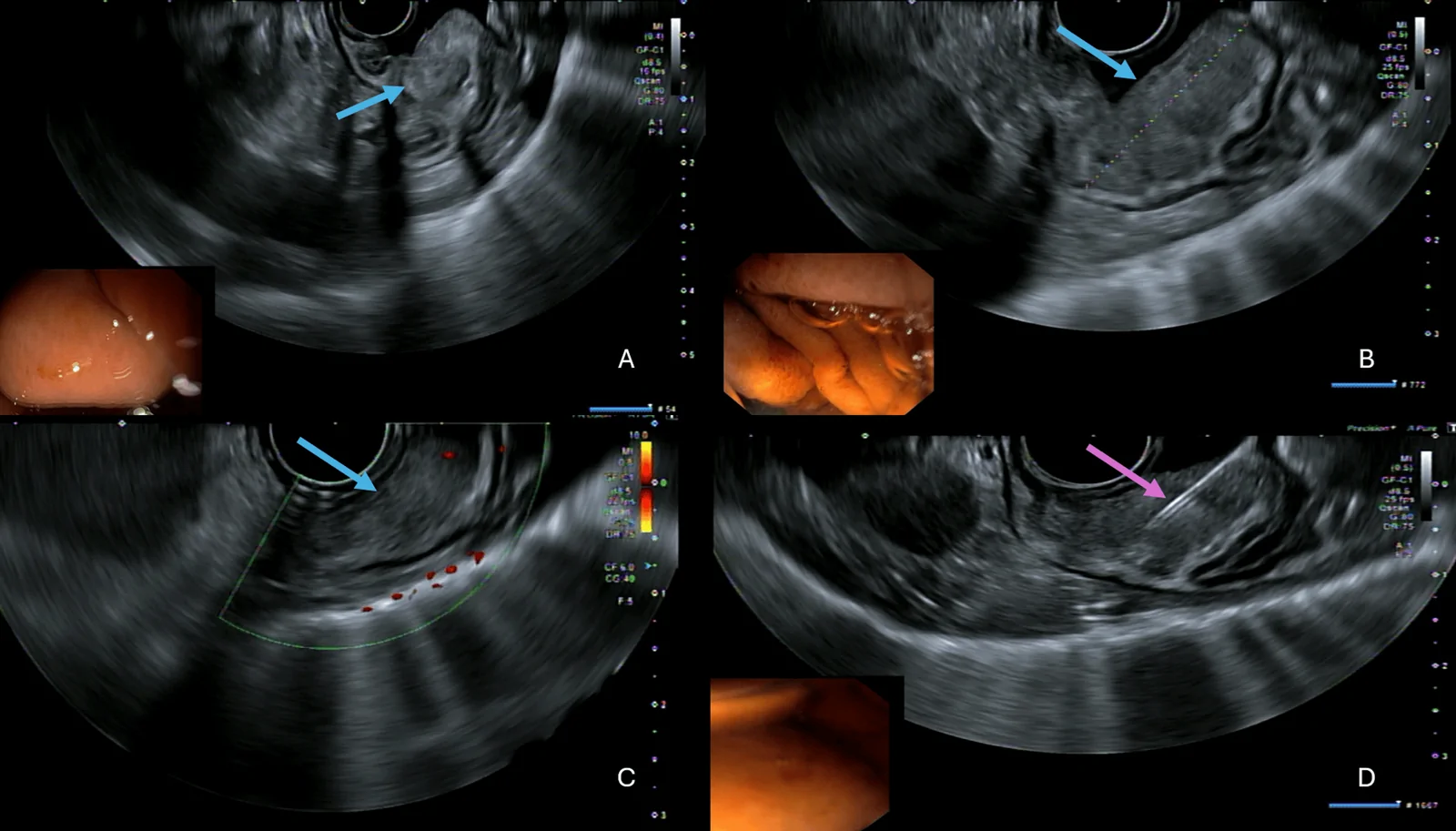
Endoscopic ultrasonographic findings of the gastric subepithelial lesion (A) Well-defined, predominantly hyperechoic submucosal lesion exhibiting mildly heterogeneous internal echostructure with small hypoechoic to anechoic areas (blue arrow). (B) Longitudinal view showing the lesion measuring approximately 25 mm, with preservation of the underlying muscularis propria (blue arrow). (C) Color Doppler examination demonstrating no significant internal vascularity (blue arrow). (D) Endoscopic ultrasound-guided needle sampling for histopathological assessment (purple arrow)

To establish a histological diagnosis, EUS-guided needle sampling was performed with two passes (Figure 3D). Tissue acquisition was technically challenging because of viscous material adhering to the needle. Additional deep bite-on-bite forceps biopsies were therefore obtained to improve tissue sampling.

Histopathological examination revealed an unremarkable superficial fundic mucosa. Within the submucosa, dilated and architecturally distorted fundic-type glands were identified without cytological atypia. Immunohistochemical staining demonstrated positivity for pepsinogen and MUC6, supporting fundic gland differentiation (Figure 4). These findings favored a benign hamartomatous or dystrophic glandular lesion, compatible with a GHIP.

**Figure 4:**
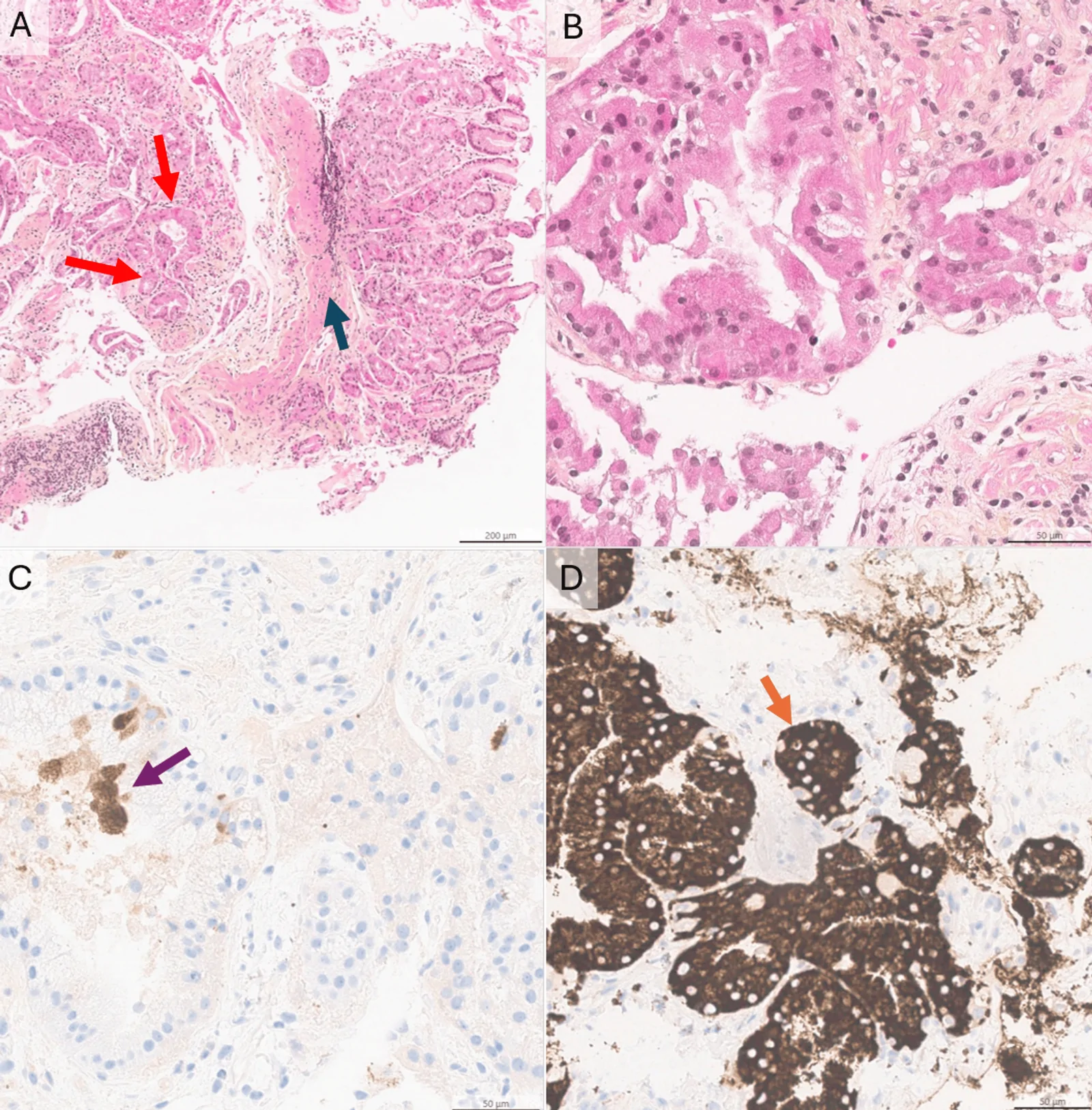
Histopathological and immunohistochemical features of the GHIP GHIP: gastric hamartomatous inverted polyp; HPS: hematoxylin-phloxine-saffron; H&E: hematoxylin and eosin (A) HPS staining at low magnification showing a submucosal proliferation of nonneoplastic gastric glands (red arrows) with intervening smooth muscle bundles (blue arrow) (scale bar: 200 µm). (B) Higher magnification demonstrating nonneoplastic fundic-type gastric glands lined by benign glandular epithelium, without cytological atypia or identifiable mitotic figures (H&E staining; scale bar: 50 µm). (C) Immunohistochemical staining showing positivity for MUC5AC in foveolar-type cells (purple arrow) (scale bar: 50 µm). (D) Immunohistochemical staining showing diffuse positivity for MUC6 in gastric glands (orange arrow) (scale bar: 50 µm).  Submucosal fundic type glands also expressed pepsinogen (not shown)

Thus, although the lesion initially raised suspicion of a GIST on conventional endoscopy and subsequently exhibited EUS features suggestive of a lipoma, histopathological and immunohistochemical findings supported a diagnosis of GHIP, highlighting the diagnostic challenges associated with this rare gastric subepithelial lesion. Following multidisciplinary team discussion, conservative management was favored over endoscopic or surgical resection, given the benign histopathological findings and the absence of cytological atypia.

## Discussion

GHIPs are benign gastric lesions characterized by the downward proliferation of foveolar, pyloric, or fundic-type glandular epithelium into the gastric submucosa, often accompanied by cystic glandular dilatation and branching smooth muscle bundles [1,3]. Their pathogenesis remains incompletely understood, although glandular displacement through defects in the muscularis mucosae and chronic inflammatory processes have been proposed as possible mechanisms [2]. Despite their benign histological nature, GHIPs represent a diagnostic challenge because they may mimic other gastric subepithelial lesions on conventional endoscopy and endoscopic and EUS [1,6].

GHIPs are frequently discovered incidentally during upper gastrointestinal endoscopy, although some patients present with abdominal discomfort, gastrointestinal bleeding, or nonspecific digestive symptoms [2]. The gastric body is the most frequently reported location (71%), followed by the fundus (18%), antrum (6%), and cardia (4%) [1]. Endoscopically, GHIPs exhibit variable appearances, ranging from sessile or pedunculated polyps to subepithelial protrusions covered by normal-appearing mucosa [1]. In the series reported by Dong et al., the lesions exhibited considerable variability in their location, size, and endoscopic appearance, contributing to difficulties in establishing a preoperative diagnosis [2]. In our patient, the lesion was identified in the gastric fundus during investigations for persistent epigastric pain, unintentional weight loss, and altered bowel habits. Conventional endoscopy revealed a broad-based, dome-shaped subepithelial protrusion with intact overlying mucosa, initially raising suspicion of a GIST.

EUS is a key diagnostic modality for characterizing gastric subepithelial lesions by identifying their layer of origin, echogenicity, internal architecture, and relationship with the muscularis propria [8,9]. GISTs generally appear as hypoechoic lesions arising from the fourth gastric wall layer, whereas lipomas typically present as well-circumscribed, homogeneous, hyperechoic lesions originating from the submucosa [8]. GHIPs, however, may exhibit variable endosonographic patterns, including heterogeneous echogenicity and intralesional cystic or anechoic areas that may reflect dilated glandular structures [1,2]. Importantly, Tanaka et al. described a GHIP with prominent internal hyperechogenicity, demonstrating that a hyperechoic appearance does not necessarily indicate a lipomatous lesion [6]. In our case, EUS findings initially favored gastric lipoma, a diagnosis further supported by the positive cushion sign observed during conventional endoscopy. However, the internal heterogeneity and small anechoic areas were less characteristic of a typical lipoma.

A comparison with previously published cases illustrates the diversity of GHIP presentations (Table 2). Ohtsu et al. reported variable gastroscopic appearances and endosonographic features, ranging from predominantly hyperechoic lesions with small anechoic components to heterogeneous lesions containing cystic spaces [1]. Similarly, Tanaka et al. described a GHIP exhibiting marked internal hyperechogenicity without posterior acoustic attenuation, highlighting its potential to mimic other subepithelial tumors [6]. Despite these differences in endoscopic appearance, histopathological examination demonstrated submucosal glandular proliferation, with immunohistochemical findings supporting gastric epithelial differentiation in the cases reported by Ohtsu et al. [1]. The clinicopathological series published by Dong et al. also emphasizes the considerable morphological variability of these lesions [2]. Our observation contributes to this spectrum by demonstrating a predominantly hyperechoic fundic submucosal lesion with subtle internal heterogeneity, initially suspected to be a GIST on conventional endoscopy and subsequently interpreted as a lipoma on EUS despite a different diagnostic suspicion at conventional endoscopy, as previously described.

**Table 2:** Comparison of previously reported gastric hamartomatous inverted polyps with the present case EUS: endoscopic ultrasonography; EMR: endoscopic mucosal resection; ESD: endoscopic submucosal dissection; M: male; F: female

Author/patient	Age/sex	Location/size	Gastroscopic findings	EUS findings	Histopathological and immunohistochemical findings	Management
Ohtsu et al. (2023), patient 2 [1]	69/M	Lower gastric body, 18 mm	Sessile subepithelial lesion with an irregular, reddish surface	Predominantly hyperechoic lesion with a small anechoic area	Well-circumscribed, lobulated submucosal proliferation of cystically dilated hyperplastic glands. MUC5AC +, MUC6+, pepsinogen-I +, focal MUC1+, CDX2+ and H+/K+ATPase +, MUC2-, Ki-67 <2%	EMR
Ohtsu et al. (2023) patient 3 [1]	62/M	Middle gastric body, 21 mm	Sessile subepithelial lesion covered by smooth, normal-appearing mucosa	Anechoic components with internal septations and hyperechoic solid areas	Cystically dilated hyperplastic gastric glands without atypia. MUC5AC+, MUC6+, pepsinogen-I+, focal CDX2+ and H+/K+ ATPase+, MUC1-, MUC2-, Ki67 2-3%	ESD
Tanaka et al. (2025) [6]	58/F	Near gastroesophageal junction, 26 mm	Lobulated subepithelial mass covered by intact mucosa	Marked internal hyperechogenicity without posterior acoustic attenuation	Inverted proliferation of gastric crypt epithelium and sparse spindle cells in a striform pattern. Initial biopsy: SMA+, DOG1-, S100-	Combined laparoscopic-endoscopic surgery
Present case	61/M	Fundus, 25 mm	Well circumscribed, broad-based subepithelial protrusion covered by intact mucosa, without ulceration or obvious NBI abnormalities; positive cushion sign	Predominantly hyperechoic, mildly heterogeneous lesion arising from the third gastric wall layer, with small hypoechoic areas and no significant internal vascularity	Submucosal proliferation of non-neoplastic fundic-type gastric glands without cytological atypia or mitotic figures. MUC5AC+, MUC6+, Pepsinogen+	Conservative management following multidisciplinary discussion

Histopathological examination is essential for establishing the diagnosis of GHIP, typically revealing ectopic gastric glands extending into the submucosa, with variable cystic dilatation and occasional intervening smooth muscle bundles [1]. Immunohistochemical positivity for MUC6 and pepsinogen supports fundic gland differentiation when interpreted in conjunction with the glandular morphology [1]. In our patient, histopathological examination demonstrated architecturally distorted, nonneoplastic fundic-type gastric glands within the submucosa, without significant cytological atypia or identifiable mitotic figures. Intervening smooth muscle bundles were also observed. Immunohistochemical analysis demonstrated MUC5AC and MUC6 expression, while additional pepsinogen positivity supported fundic gland differentiation. These findings were compatible with GHIP.

In 1947, Scott and Payne reported a tumorlike gastric lesion characterized by diffuse cystic hyperplasia within the gastric submucosa, an early description of what is now recognized as gastritis cystica profunda (GCP) [4]. GCP is a rare condition that represents an important histopathological differential diagnosis of GHIP, as both entities may exhibit cystically dilated gastric glands extending into the submucosa [4,10]. GCP is more commonly associated with previous gastric surgery or endoscopic interventions, including polypectomy [4]. Differentiation between GHIP and GCP relies on the overall glandular architecture, the pattern of submucosal glandular proliferation, and the distribution of associated smooth muscle bundles [1]. However, considerable histological overlap may exist between these entities, and their distinction should be based on the overall morphological and clinical context rather than on a single histological feature.

Although GHIPs are generally benign, occasional associations with epithelial dysplasia and gastric adenocarcinoma have been reported [3,7]. Kono et al. suggested that p53 alterations might contribute to neoplastic transformation in some GHIPs; however, this hypothesis remains insufficiently substantiated [11]. Current evidence is insufficient to establish their intrinsic premalignant potential, underscoring the importance of careful histopathological assessment.

Management remains poorly standardized. Endoscopic resection may provide both definitive treatment and complete histological assessment [2]. Current European Society of Gastrointestinal Endoscopy (ESGE) and American Gastroenterological Association (AGA) recommendations emphasize tissue acquisition and individualized management of gastric subepithelial lesions according to size, suspected diagnosis, and malignant potential [8,9]. Advanced endoscopic resection techniques may be considered in selected cases [12]. In our patient, EUS-guided fine-needle biopsy followed by deep bite-on-bite biopsies provided adequate tissue for histopathological and immunohistochemical examination, supporting the diagnosis of GHIP and allowing conservative management without the need for immediate endoscopic or surgical resection. 

Our case highlights the diagnostic challenges of GHIP presenting as a predominantly hyperechoic gastric subepithelial lesion. Initially suspected to be a GIST on conventional endoscopy and subsequently a lipoma on EUS, the lesion was ultimately considered compatible with GHIP based on histopathological and immunohistochemical examination. This observation emphasizes the importance of considering GHIP in the differential diagnosis of atypical hyperechoic gastric subepithelial lesions.

## Conclusions

GHIP is a rare benign lesion that may mimic other gastric subepithelial tumors. Our case illustrates its unusual predominantly hyperechoic appearance on EUS, initially suggesting a gastric lipoma despite an earlier suspicion of a GIST. Histopathological examination, supported by immunohistochemistry, was essential in supporting the diagnosis of GHIP. GHIP should therefore be considered in the differential diagnosis of atypical hyperechoic gastric subepithelial lesions to ensure accurate diagnosis and appropriate management.

## References

[1] Ohtsu T, Takahashi Y, Tokuhara M (2023). Gastric hamartomatous inverted polyp: report of three cases with a review of the endoscopic and clinicopathological features. DEN Open.

[2] Dong N, Meng F, Yue B, Hou J (2024). Clinicopathologic and endoscopic characteristics of ten patients with gastric hamartomatous inverted polyp: a single center case series. BMC Gastroenterol.

[3] Park G, Kim J, Lee SH, Kim Y (2023). Large gastric hamartomatous inverted polyp accompanied by advanced gastric cancer: a case report. World J Clin Cases.

[4] De Stefano F, Graziano GM, Viganò J (2023). Gastritis cystica profunda: a rare disease, a challenging diagnosis, and an uncertain malignant potential: a case report and review of the literature. Medicina (Kaunas).

[5] Effenberger M, Steinle H, Offner FA, Vogel W, Millonig G (2014). Holes in gastric mucosa in upper gastrointestinal endoscopy. Eur J Gastroenterol Hepatol.

[6] Tanaka H, Oura S, Kataoka N (2025). Gastric hamartomatous inverted polyps have high internal echoes: a case report. Radiol Case Rep.

[7] Okamura T, Iwaya Y, Nagaya T, Muranaka F, Ota H, Umemura T (2022). Gastric adenocarcinoma arising from hamartomatous inverted polyp during 8-year follow-up. DEN Open.

[8] Deprez PH, Moons LM, OʼToole D (2022). Endoscopic management of subepithelial lesions including neuroendocrine neoplasms: European Society of Gastrointestinal Endoscopy (ESGE) Guideline. Endoscopy.

[9] Sharzehi K, Sethi A, Savides T (2022). AGA clinical practice update on management of subepithelial lesions encountered during routine endoscopy: expert review. Clin Gastroenterol Hepatol.

[10] Lee SJ, Park JK, Seo HI (2013). A case of gastric inverted hyperplastic polyp found with gastritis cystica profunda and early gastric cancer. Clin Endosc.

[11] Kono T, Imai Y, Ichihara T (2007). Adenocarcinoma arising in gastric inverted hyperplastic polyp: a case report and review of the literature. Pathol Res Pract.

[12] Pimentel-Nunes P, Libânio D, Bastiaansen BA (2022). Endoscopic submucosal dissection for superficial gastrointestinal lesions: European Society of Gastrointestinal Endoscopy (ESGE) Guideline-update 2022. Endoscopy.

